# A prospective development and evaluation of a 2D convolutional neural network-based auto-segmentation model for cervical cancer radiotherapy

**DOI:** 10.1186/s43046-026-00382-7

**Published:** 2026-07-15

**Authors:** Sidharth Satish Menon, Mahak Gupta, Aakriti Bhardwaj, Srinidhi G Chandraguthi, Jayashree NP, Umesh Velu, Anshul Singh, Ankita Mehta, Srinivasan Vijayakumar, Lavanya Gurram, Chaitra Raghuram, Roshan David Jathanna, Shirley Lewis

**Affiliations:** 1 Department of Radiation Oncology, Kasturba Medical College, Manipal Academy of Higher Education, Manipal, India; 2 Department of Radiology, Ochsner Clinic Foundation, New Orleans, LA USA; 3 Department of Radiation Oncology, BC Cancer Agency-Abbotsford, University of British Columbia, Vancouver, Canada; 4 AIRONC Healthcare Technologies Private Ltd, Manipal, India; 5 Manipal Institute of Technology, Manipal Academy of Higher Education, Manipal, India

**Keywords:** Radiotherapy, Cervical malignancy, Artificial Intelligence, Deep learning, Machine Learning, Convolutional Neural Network

## Abstract

**Background:**

Accurate delineation of target volumes and organs at risk (OAR) is essential in radiotherapy planning for cervical cancer. Deep learning (DL)-based auto-segmentation has the potential to improve contouring efficiency and workflow. This study reports the prospective development and internal validation of a DL-based auto-segmentation model- Deep contour (DC) for cervical cancer radiotherapy.

**Methods:**

In this prospective single-institution study, a 2-dimensional convolutional neural network based on the LinkNet architecture, DC, was trained on 190 computed tomography (CT) datasets for abdominal and pelvic OARs and 90 cervical cancer datasets for target volumes. Independent validation was performed on 20 CT datasets. Model performance was evaluated using dice similarity coefficient (DSC), Jaccard Index (JI), 95th percentile Hausdorff distance (HD95), average symmetric surface distance (ASSD), and surface dice coefficient (NSD). Expert internal and external radiation oncologists qualitatively explored clinical acceptability using a Likert scale, and segmentation time was compared with manual contouring.

**Results:**

The DC demonstrated the greatest geometric performance for the femur (DSC 0.92 ± 0.03; NSD 0.94 ± 0.04), bowel bag (DSC 0.89 ± 0.03; NSD 0.77 ± 0.12), and bladder (DSC 0.88 ± 0.14; NSD 0.87 ± 0.12). Among the target volumes, the inguinal nodal clinical target volume (CTVn_inguinal) achieved the greatest agreement (DSC 0.77 ± 0.04; NSD 0.76 ± 0.07). Moderate performance was observed for the rectum (DSC 0.75 ± 0.16), liver (DSC 0.73 ± 0.21), and pelvic nodal clinical target volume (CTVn_pelvis) (DSC 0.60 ± 0.10), whereas lower performance was observed for anatomically complex structures such as the duodenum, anal canal, common bile duct, pancreas, and pelvic vessels. Clinical evaluation of two cases revealed a Likert score of III-IV for key pelvic organs, such as the bladder, femur, pelvic bowel bag, rectum, and sigmoid. Auto-segmentation significantly reduced the segmentation time from 77 min to 5 s per dataset (*p* < 0.001).

**Conclusions:**

This prospective validation demonstrates that DC auto-segmentation model can achieve acceptable geometric performance congruent across multiple abdominal and pelvic OARs and reasonable geometric performance for the elective inguinal CTV volume. Further validation on larger datasets and evaluation of clinical workflow integration are warranted.

**Registry:**

CTRI, TRN: CTRI/2024/02/063055, Registration date: February 22, 2024.

**Supplementary Information:**

The online version contains supplementary material available at 10.1186/s43046-026-00382-7.

## Background

Cervical cancer remains a major global health burden and one of the leading causes of mortality in low-medium Human Development Index countries, with a global average of 7.1 deaths per 100, 000 women deaths [1]. Often presenting in the locally advanced stage, definitive chemoradiotherapy followed by brachytherapy is the standard of care [2]. In countries such as India with resource-constrained institutions and a high disease burden, the overall time to treatment initiation (TTI) is 20 days, and patients requiring radiotherapy may wait up to 27.5 days [3]. The timely initiation and uninterrupted delivery of radiotherapy are critical to treatment outcomes. Prolonged overall treatment time (OTT) has been consistently associated with reduced pelvic control and survival because of accelerated repopulation during treatment [4, 5]. Therefore, minimising delays at all stages of radiotherapy planning and delivery is essential for achieving optimal clinical outcomes. Accurate delineation of target volumes and organs at risk (OAR) is fundamental and the rate-limiting step in radiotherapy planning. Segmentation guides dose optimisation and directly influences tumour coverage and normal tissue sparing. However, manual segmentation is a labour-intensive and time-consuming process that requires clinical expertise and knowledge and is subject to inter-and intra-observer variability. A recent survey by the Royal College of Radiology reported a median time of 85 min for manual segmentation, with gynaecological cases requiring a median of 120 min for segmentation, not including the time required for peer review [6]. This can contribute to workflow bottlenecks and treatment planning delays. Such delays may significantly impact the timely initiation and completion of radiotherapy in high-volume centers with resource constraints, thereby affecting adherence to the recommended OTT.

The emergence of deep learning (DL)-based convolutional neural networks (CNN) and their applications in medical imaging, has enabled effective auto-segmentation in pelvic cancers such as prostate and rectum [7, 8]. The National Institute for Health and Care Excellence (NICE) advocates for the use of AI-based technology in various aspects of RT planning, especially auto-segmenting, highlighting potential cost and time savings [9]. Vendor-based DL models often employ U-Net and variant architectures as integrated solutions within the treatment planning system (TPS) allowing seamless end-to-end segmentation. However, commercial models do not allow training and validation by the end-user and are limited in their transparency regarding training datasets and parameters [10]. The NRG Oncology Working Group recommends guiding the evaluation and clinical implementation of commercial and research-based auto-segmentation solutions [11]. Model evaluation is carried out through quantitative measurements of volume and distance, a comparison of dosimetry and time-savings, with the critical judgment of clinical value by physicians [12, 13]. Few studies in literature focus on training DL models for both the OARs and target volumes. While the most commonly used evaluation metric is geometric distance, only 19% and 16% of the studies assessed subjective evaluation and time-savings, respectively [12]. This study aimed to develop and prospectively validate a DL-based auto-segmentation model for target volumes of cervical cancer and OARs of abdomen and pelvis and to comprehensively evaluate the model for geometric accuracy, clinical acceptability and impact on time-saving.

## Methods

### Study workflow

**Fig. 1:**
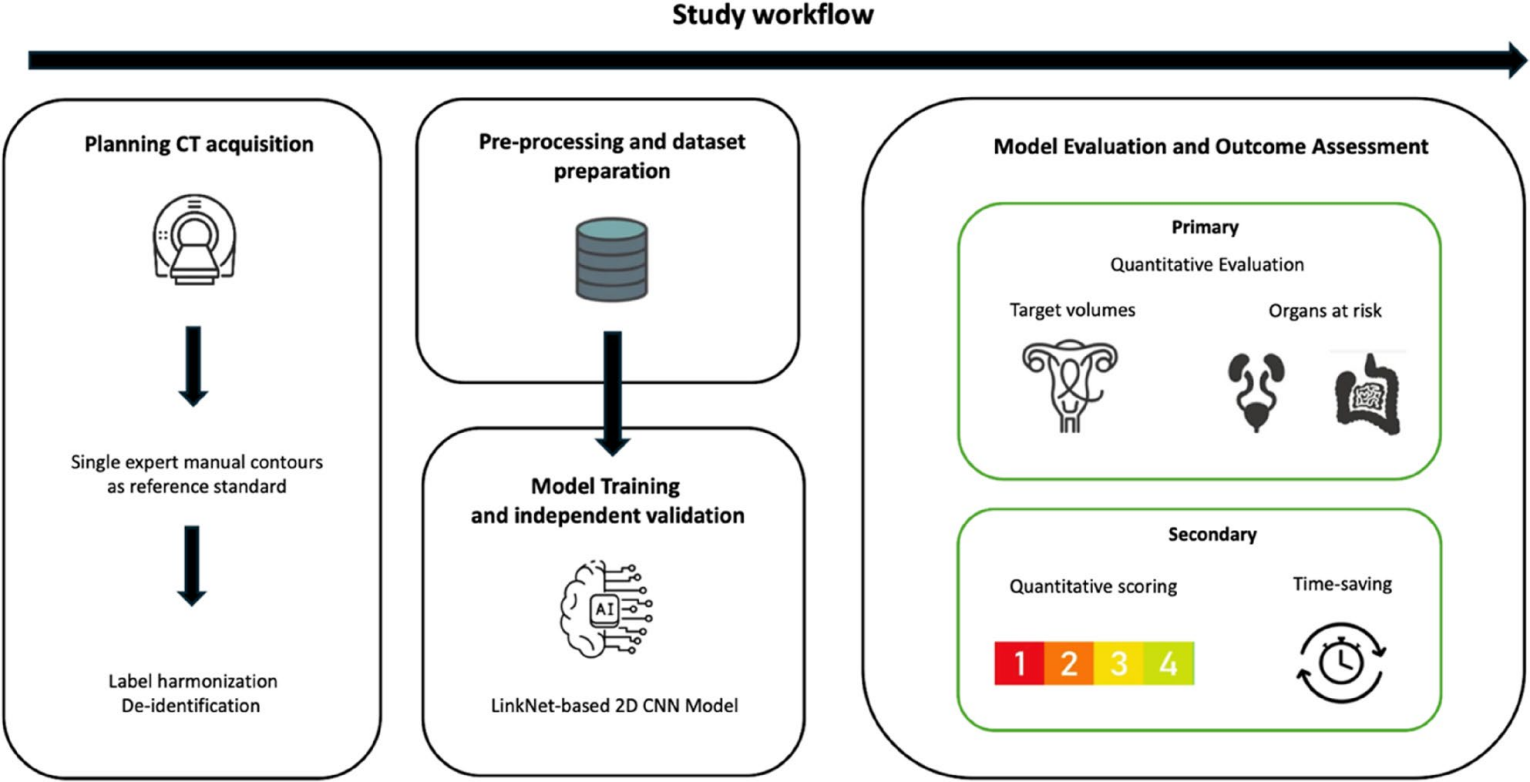
Study workflow

This prospective observational study was conducted at a single tertiary center from October 2022 – April 2025. This study was approved by the Institutional Ethics Committee (Approval No.ECR/191/Inst/KL/2013/RR-19) and is registered in clinical trials registry of India (CTRI/2024/02/063055). The study workflow consists of the following steps: acquisition of input CT image datasets, manual segmentation of the OARs and target volumes and standardisation of datasets for ingestion for the DL model; ‘DEEP CONTOUR^TM^’ (DC). Training was conducted in a secure server followed by independent validation for outcome assessment (Fig. 1). The primary endpoint was quantitative assessment using volume overlap, distance agreement and surface overlap metrics. Secondary endpoints included qualitative clinical acceptability assessed using the Likert scale by expert oncologists and comparison of contouring time between manual and automated segmentation.

There were 190 and 90 de-identified DICOM datasets for OARs and for target volumes, respectively, with no subsequent investigator access to patient-identifiable information during model development and evaluation. The planning computed tomography (CT) scans and radiotherapy-related data (DICOM files, structure sets, plan parameters, dose volume histograms, and dose constraints) were retrieved from institutional repository. Non-repetitive retrospective and prospective planning CT scans of abdomen and pelvic cancers acquired under the institutional protocol and eligible for external beam radiotherapy with curative or palliative intent were selected and curated for anonymization. Pelvic cancer cases planned for curative radiotherapy were acquired with a full bladder in the supine position with or without contrast using the Phillips big bore CT [14]. Datasets with incomplete imaging, missing structure sets or corrupted DICOM metadata were excluded prior to model training and validation; therefore, they did not influence model development and evaluation. The OARs and target volumes were labeled according to the study protocol and manually checked in accordance with established international contouring guidelines [15–17]. Expert-verified manual contours were chosen as the reference standard because physician-delineated contours remain the accepted clinical gold standard for radiotherapy planning. The target volumes included the primary clinical target volume (CTVp), the elective pelvic lymphatic drainage regions (CTVn_pelvis), and the inguinal region (CTVn_inguinal). All the OARs of the abdomen and pelvis were segmented. The observers did not have access to the model outputs during the manual contour generation for the validation set. The adequacy of anonymization was verified by a data scientist and subsequently uploaded to Google Cloud for training of the model [18].

### Preprocessing

The model input was in DICOM format, with standard dimensions of 512 × 512 pixels and varying z-dimensions across the scout views of the acquired image. The voxel size was not interpolated or otherwise processed. Data augmentation was employed during training to improve the generalisation of the model. The augmentation pipeline began with a random center crop, with crop sizes varying between 256 × 256 and 300 × 300 pixels. This was followed by resizing the image to a fixed resolution of 512 × 512 pixels. Then, a Gaussian blur was applied, and random brightness and contrast adjustments were introduced, each with a probability of 0.5. Additionally, a No-Operation (NoOp) transformation was applied with a probability of 0.5 to both the input image and the corresponding ground truth mask, allowing some samples to bypass further stochastic augmentations while ensuring consistent application to both the image and the label.

### Deep learning architecture

The DC segmentation model used in this study was based on the *LinkNet* architecture, specifically employing the *LinkNet34* configuration, which is an efficient encoder-decoder network designed for semantic segmentation. The architecture features direct skip connections between the corresponding encoder and decoder layers, enabling the transfer of fine-grained spatial information and improving the reconstruction accuracy. The encoder component used a ResNet 34 backbone comprising five stages, resulting in an effective encoder depth of five. This backbone was pretrained on the *ImageNet* dataset to leverage transfer learning. For downsampling, max pooling operations with a kernel size of 2 × 2 were employed, whereas upsampling was performed using convolutions with a kernel size of 3 × 3. A 2-dimensional (2D) learning approach was implemented, with individual axial slices from the volumetric CT as input. As training with 3D images would require substantially higher graphics processing unit (GPU) requirements, using a 2D approach enables efficient batch processing and stable training within the constraints of available computational resources. No ensembling techniques were employed.

### Training and validation of models

DC was trained on a server equipped with dual Intel Xeon Gold 6140 CPU @ 2.30 GHz, with each CPU featuring 18 physical cores and 36 threads, resulting in 36 CPU cores and 72 threads. The system utilized an NVIDIA A100 80GB PCIe GPU with 251GB RAM and 80GB of memory dedicated bandwidth. The installed NVIDIA driver was 570.133.20, which supports CUDA version 12.8. The input images were processed using a batch size of eight. The Adam optimizer with an initial learning rate of 0.001 was employed.To facilitate a gradual learning rate decay, a step-based learning rate scheduler (torch.optim.lr_scheduler.StepLR) was used, reducing the learning rate by a factor of 0.95 every three epochs. The primary loss function adopted for training was the Cross-Entropy Loss. Model checkpointing was implemented to ensure the efficient use of computational resources and to allow recovery from intermediate training states. All experimental runs, including the training metrics and hyperparameters, were logged and tracked using the Weights & Biases platform, enabling reproducibility and detailed analysis. The final model was selected at the epoch where the overall validation DSC curve plateaued above approximately 0.8, indicating convergence.

### Model evaluation

DC was evaluated in an independently curated validation set separate from the training set. Quantitative evaluation was performed using the Dice similarity coefficient (DSC), Jaccard Index (JI), 95th percentile Hausdorff distance (HD95), average symmetric surface distance (ASSD), and surface dice coefficient (NSD). The DICOM containing the model output was imported into the Monaco [19] TPS and 3D slicer [20] for clinical evaluation. An unblinded clinical evaluation of the auto-segmented volumes were conducted and the volumes were ranked by five internal experts with varying levels of experience and two external experts on a Likert scale of 1–4, indicating a range of clinical acceptability [21]. The time required to manually segment each volume and the composite time required to complete each validation dataset were recorded for comparison with DC.

### Data analysis

As there are no clear guidelines regarding the recommended number of training and validation sets [22], we used convenience sampling. The training set consisted of 190 CT datasets of abdominal and pelvic organs and 90 CT datasets for cervical cancer target volumes. The DICOM datasets were partitioned at the patient level, ensuring that no imaging dataset from the same patient appeared in more than one subset. Independent validation was performed using 20 separate CT datasets not included in the training cohort. The imaging characteristics and demographic details were descriptively reported. The geometric agreement in terms of the quantitative metrics for the OARs and target volumes is reported as mean and standard deviation. The qualitative evaluation using Likert scores was summarised descriptively. The difference in contouring time between manual and automated segmentation was evaluated using paired statistical analysis for each organ. Assuming non-normal data, the Mann-Whitney U test was used, with statistical significance defined using a two-sided p-value threshold of < 0.05. All statistical analyses were performed on Jamovi [23, 24]. As only de-identified data were used in the study, the patient consent was waived. The study reporting complies with the MI-CLAIM checklist (Supplementary File 1).

## Results

A total of 190 CT datasets were successfully ingested by DC for training and 20 CT datasets were used for validation. The demographic, clinical characteristics and input image details are summarised in Table 1. The distribution of the primary sites used for OAR training and validation can be found in Table 2.

**Table 1:** Demographic and imaging characteristics of the training and validation datasets used for model development

Characteristic	Training set OARs (*n* = 190) *N* (%)	Training set target volumes (*n* = 90) *N* (%)	Validation set OARs (*n* = 20) *N* (%)	Validation set target volumes (*n* = 20) *N* (%)
Gender				
Male	60 (31.6%)	-	4(20%)	-
Female	130 (68.4%)	90 (100%)	16(80%)	20 (100%)
Slice thickness (mm)				
2 mm	4 (2.1%)	-	-	-
3 mm	86 (45.2%)	26 (28.88%)	20(100%)	13 (65%)
5 mm	100 (52.6%)	64 (71.12%)	-	07 (35%)
Contrast				
Yes	51 (26.84%)	32 (35.55)	08 (40%)	12 (60%)
No	139 (73.1%)	58 (64.45%)	12 (60%)	08 (40%)

**Table 2:** Distribution of cases for training and validation cohorts for organ at risk

Training set	*N* = 190
Carcinoma breast	5
Carcinoma cervix	80
Carcinoma Rectum	38
Carcinoma Prostate	9
Metastatic case	26
Carcinoma endometrium	4
Carcinoma anal canal	4
Miscellaneous	14
Multiple myeloma	5
Carcinoma stomach	3
Carcinoma bladder	2

Validation set	*N* = 20
Rectal malignancy	3
Cervical malignancy	11
Carcinoma Penis	1
Carcinoma Anal canal	1
Metastatic case	2
Carcinoma stomach	1
Synchronous Rectal and prostate malignancy	1

**Fig. 2:**
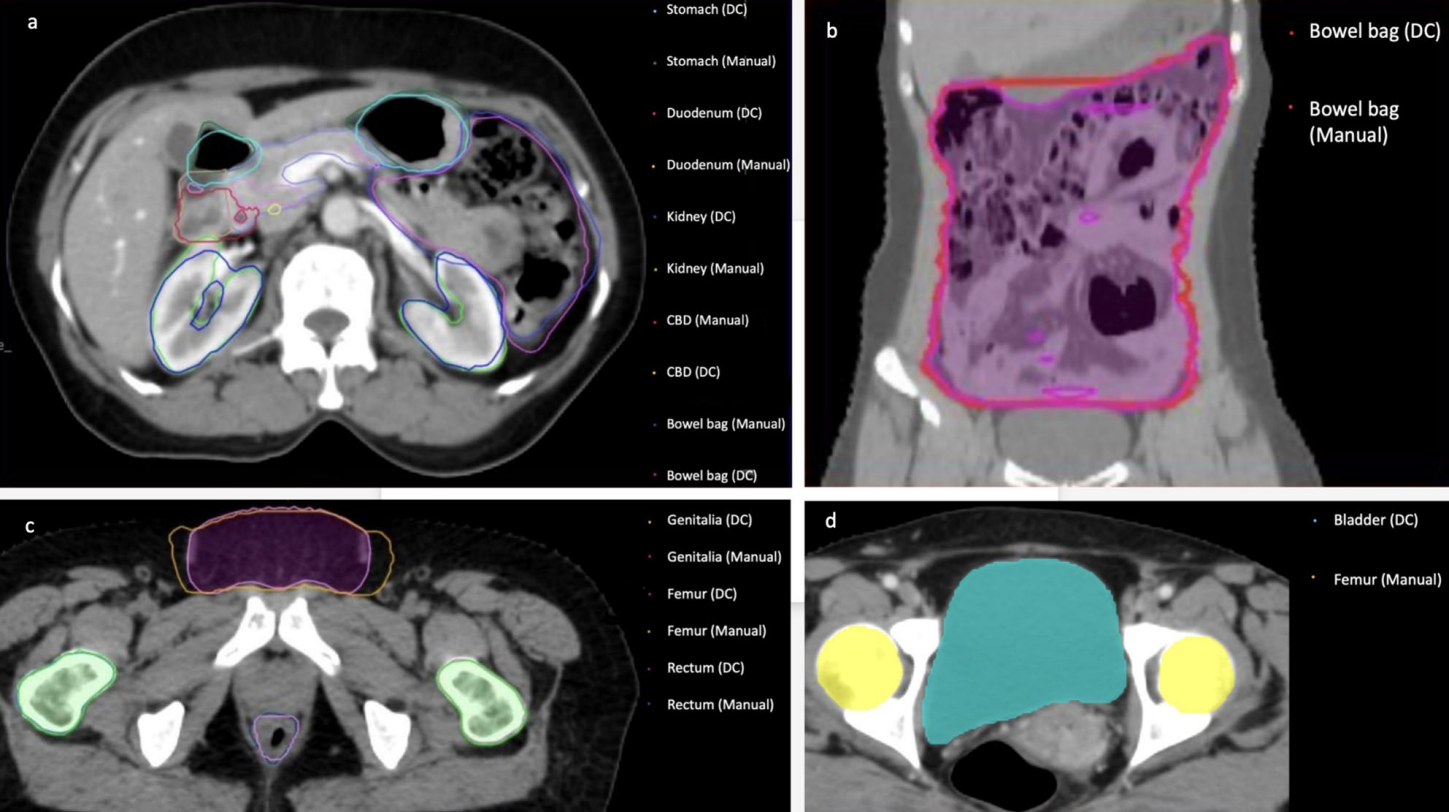
Representative visual comparison of auto-segmented and manually delineated abdominal and pelvic organs at risk; **a** Axial abdominal slice demonstrating overlay of manual and auto-segmented contours for upper abdominal organs. **b** Coronal abdominal view illustrating cranio-caudal contour conformity of auto-segmented and manually segmented bowel bag volume. **c** Axial pelvic slice showing overlap of manual and auto-segmented contours for pelvic organs. **d** Representative auto-segmentation output for pelvic organs without manual overlay

**Fig. 3:**
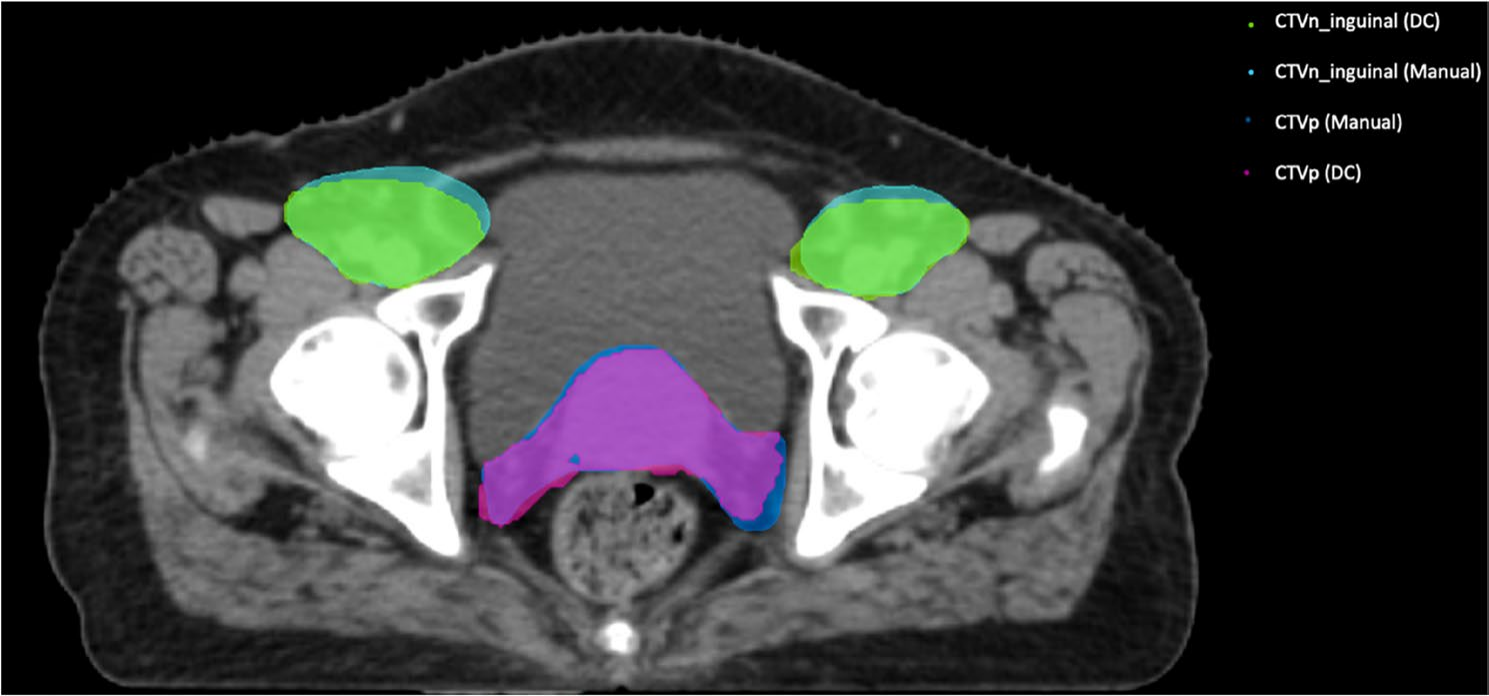
Representative visual comparison of auto-segmented and manually delineated pelvic target volumes; Axial pelvic slices demonstrating overlap between auto-segmented and manual contours for primary clinical target volume (CTVp) and inguinal nodal clinical target volume (CTVn_inguinal)

Representative qualitative comparisons between manual and auto-segmented structures are illustrated in Figs. 2 (a-d) and 3.

In the abdominal region, substantial spatial agreement was observed between manual and auto-segmented contours for kidneys, stomach and bowel bag, while duodenum and CBD show poorer overlap. In the pelvis, good concordance was observed for bilateral femur and genitalia, while the auto-segmented external genitalia overextended laterally. As shown in Fig. 3, good concordance was observed for the inguinal nodal volume and primary CTV volume.

### Quantitative evaluation

**Fig. 4:**
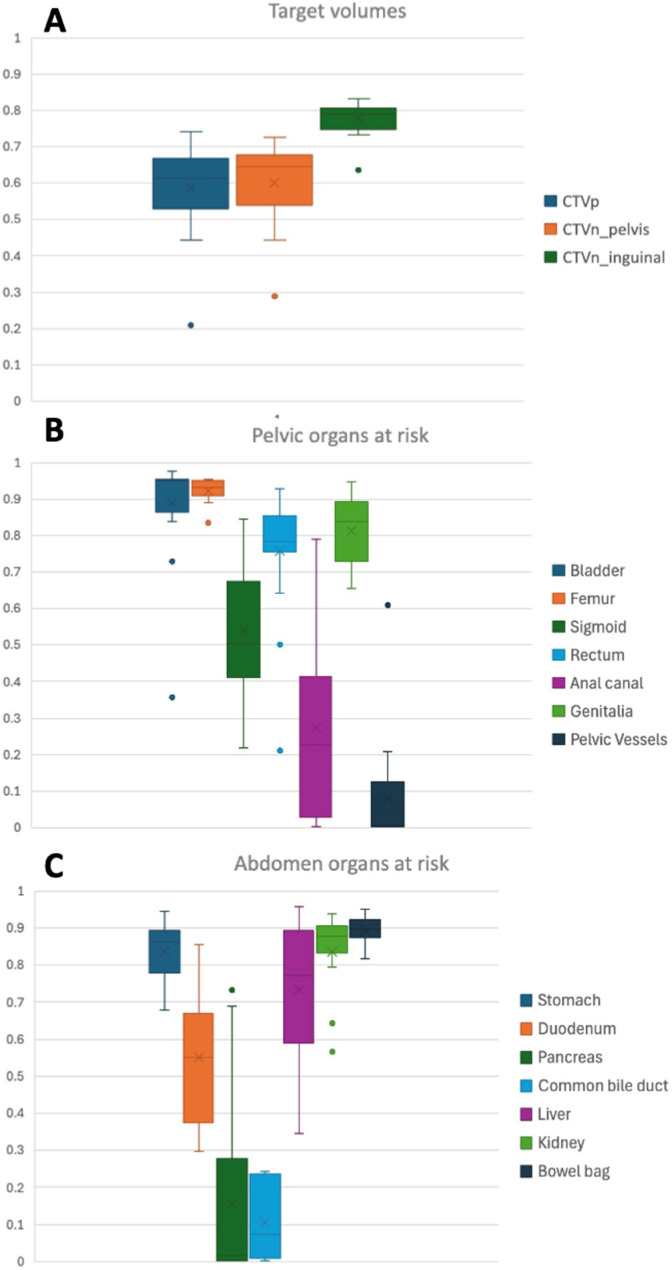
Distribution of Dice Similarity Coefficient (DSC) values for auto-segmentation of target volumes and organs at risk; Boxplots illustrating DSC values for auto-segmentation performance in the validation dataset. Panels show (**A**) target volumes, (**B**) abdominal organs at risk, and (**C**) pelvic organs at risk. Boxes represent interquartile range, center line represents median, whiskers represent range, and points represent outliers. Higher DSC indicates better agreement between manual and automated contours

DC was quantitatively evaluated using overlap, distance and surface-based geometric measures. Among the target volumes, the highest performance was observed for CTV inguinal volume with a DSC_mean_ of 0.77 ± 0.04, JI of 0.64 ± 0.06, NSD of 0.76 ± 0.07, HD95 of 36.5 ± 36.7 and ASSD of 4.57 ± 1.96. The CTVn_pelvis volume demonstrated a moderate geometric agreement [DSC_mean_ of 0.6 ± 0.1, JI of 0.43 ± 0.1, NSD of 0.71 ± 0.14, HD95 of 66.6 ± 23.8 and ASSD of 10.6 ± 10.4], while CTVp showed lower geometric overlap and boundary agreement scores. Among the OARs, the highest agreement among all metrics was obtained for femur [DSC_mean_ of 0.92 ± 0.03, JI of 0.86 ± 0.05, NSD of 0.94 ± 0.04, HD95 of 7.17 ± 7.09 and ASSD of 1.29 0.66] followed by the bowel bag [DSC_mean_ of 0.89 ± 0.03, JI of 0.86 ± 0.05 NSD of 0.77 ± 0.12, HD95 of 16.7 ± 29.1 and ASSD of 3.21 ± 3.35] and bladder [DSC_mean_ of 0.88 ± 0.14, JI of 0.81 ± 0.05, NSD of 0.87 ± 0.12, HD95 of 14.8 ± 16.2 and ASSD of 2.41 ± 2.29]. Reasonable agreement was observed for the stomach, kidneys, genitalia, whereas moderate agreement with larger HD95 and ASSD values was observed for the rectum and liver, followed by duodenum and sigmoid. Smaller and tubular structures such as CBD and pelvic vessels in contrast, performed the poorest. The detailed geometric performance scores for all target volumes and OARs are summarised in Table 3 and the DSC scores are illustrated in Fig. 4.

**Table 3:** Quantitative performance of DC on validation datasets for target volumes and organs at risk

Target Volume	DSC	JI	HD95	NSD	ASSD
CTVp	0.58(± 0.12)	0.42(± 0.11)	98.4(± 18.5)	0.66(± 0.1)	19.5(± 10.3)
CTVn_pelvis	0.6(± 0.1)	0.43(± 0.1)	66.6(± 23.8)	0.71(± 0.14)	10.6(± 10.4)
CTVn_inguinal	0.77(± 0.04)	0.64(± 0.06)	36.5(± 36.7)	0.76(± 0.07)	4.57(± 1.96)
OARs					
Stomach	0.83(± 0.07)	0.72(± 0.11)	18.5(± 24.9)	0.84(± 0.11)	2.33(± 1.61)
Duodenum	0.55(± 0.16)	0.39(± 0.17)	27.1(± 17.2)	0.71(± 0.13)	4.76(± 2.28)
Pancreas	0.15(± 0.26)	0.11(± 0.20)	56.3(± 44.6)	0.24(± 0.33)	21.7(± 19.3)
CBD	0.1(± 0.11)	0.05(± 0.06)	51.5(± 24.4)	0.35(± 0.22)	15(± 10.4)
Liver	0.73(± 0.21)	0.61(± 0.24)	59.1(± 70.5)	0.64(± 0.25)	9.16(± 17.6)
Kidneys	0.83(± 0.11)	0.73(± 0.15)	26.2(± 46.8)	0.86(± 0.11)	6.49(± 16.1)
Bowel bag	0.89(± 0.03)	0.81(± 0.05)	16.7(± 29.1)	0.77(± 0.12)	3.21(± 3.35)
Bladder	0.88(± 0.14)	0.82(± 0.17)	14.8(± 16.2)	0.87(± 0.12)	2.41(± 2.29)
Femur	0.92(± 0.03)	0.86(± 0.05)	7.17(± 7.09)	0.94(± 0.04)	1.29(± 0.66)
Sigmoid	0.54(± 0.17)	0.39(± 0.17)	51.1(± 23.2)	0.64(± 0.16)	9.36(± 5.29)
Rectum	0.75(± 0.16)	0.63(± 0.17)	17.2(± 23.5)	0.82(± 0.11)	3.49(± 2.65)
Anal canal	0.27(± 0.23)	0.18(± 0.18)	71.1(± 66)	0.48(± 0.28)	23.1(± 31)
Genitalia	0.81(± 0.09)	0.69(± 0.13)	16.5(± 16.7)	0.83(± 0.07)	2.49(± 1.36)
Pelvic vessels	0.07(± 0.15)	0.04(± 0.01)	83.9(± 54.6)	0.18(± 0.2)	27.8(± 14.2)

*DSC* Dice similarity coefficient, *JI* Jaccard index, *HD95* 95th percentile Hausdorff distance, *NSD* Normalized surface Dice, *ASSD* Average symmetric surface distance, *CTVp* Primary clinical target volume, *CTVn_pelvis* Pelvic nodal clinical target volume, *CTVn_inguinal* Inguinal nodal clinical target volume, *CBD* Common bile duct

### Clinical evaluation

An open, subjective evaluation was conducted on two randomly selected independent validation datasets by five internal and two external experts using a structured Likert scale to assess the clinical acceptability of the contours. The oncologists were not exposed to the manually segmented volumes or the geometric performance. For validation case one, the internal experts demonstrated complete agreement and deemed the common bile duct (CBD), rectum, femur and bladder and pelvic bowel bag to be clinically acceptable without edits (IV). The liver and pancreas received the lowest score and were deemed clinically unacceptable (I). The external experts assigned the highest score (IV) to femur, and demonstrated complete agreement in clinically accepting kidneys, pelvic bowel bag, bladder, sigmoid and rectum with minor edits (III).

For validation case two, the internal experts demonstrated complete agreement in clinically accepting the abdominal bowel bag with no edits (IV). No organs were deemed clinically unacceptable (I) by the internal experts, whereas one external expert found the CBD unacceptable (I). The abdominal bowel bag, liver, stomach and duodenum were found to be clinically acceptable with minor edits (III) by the external experts. When Likert scores III and IV were grouped as clinically acceptable, complete agreement was observed for kidneys, bladder, sigmoid and pelvic bowel bag auto-segmentations. Across the two cases, clinical acceptance rates exceeded 90% for the stomach, duodenum, abdominal bowel bag, and rectum. Acceptance rates ranged from 80 to 89% for the pancreas, CBD, anal canal, and external genitalia, whereas lower acceptance was observed for the femur (78.6%) and liver (64.3%). In this preliminary qualitative assessment conducted on two validation cases, the overall clinical acceptance rate (Likert score of III-IV) was 93.5% among internal experts and 82.1% among external experts. Notably, the liver was deemed clinically unacceptable by all experts in validation case one. During the qualitative assessment, the experts made the consensus decision to evaluate the bowel bag separately as an abdominal or a pelvic component because of the significant difference in contouring between these regions. The detailed Likert scale scores can be found in Supplementary File 2.

### Time saving

Manual segmentation required a mean time of 65 min for the OARs and 11 min for the target volumes, resulting in a total average contouring time of 77 min. Among the OARs, liver and bowel bag required the longest time, with a mean duration of 11 min each. In contrast, auto-segmentation was completed in 5 s per dataset under laboratory conditions. The difference in average segmentation time between the manual and automated approaches was statistically significant (Mann-Whitney U = 0.00, *p* < 0.001; Table 4).

**Table 4:** Comparison of time required for manual segmentation and auto-segmentation of target volumes and organs at risk

Structure	Manual segmentation (min: sec)	Auto-segmentation DC (min: sec)
Stomach	08:54	
Duodenum	04:05	
Pancreas	04:29	
CBD	01:04	
Liver	11:24	
Kidneys	02:59	
Bowel bag	11:19	
Bladder	02:40	
Sigmoid	03:40	
Rectum	02:23	
Anal canal	01:06	
Femur	04:16	
Genitalia	04:06	
Pelvic vessels	04:08	
GTVp	01:15	
CTVp	03:30	
CTVn_pelvis	05:16	
CTVn_inguinal	0:55	
Total	77:48	00:05

## Discussion

DC is trained on real-world clinical datasets, comprising 190 abdominopelvic cases for OARs segmentation and 90 cervical cancer cases for target volumes and subsequently validated on 20 CT datasets. Quantitative performance was favourable for several large organs with distinct anatomical boundaries, including the femur, bowel bag, bladder, kidneys, stomach, and genitalia, Moderate performance was observed for rectum, liver and the CTV inguinal volume, whereas anatomically complex structures such as the duodenum, sigmoid, anal canal, pancreas, CBD and pelvic vessels, CTVp and CTVn_pelvis demonstrated lower performance. On qualitative clinical evaluation of two datasets from the validation cohort, the internal experts demonstrated good agreement with most organs requiring minor or no edits; however, one to two experts indicated that the duodenum, sigmoid, rectum and anal canal volumes required major edits. DC demonstrated substantial time-savings of over one hour compared to manual segmentation.

The present study employed a 2D LinkNet34 architecture with a pretrained ResNet-34 encoder, selected for its computational efficiency and direct skip connections that preserve spatial information relevant to organ boundary delineation [25]. This approach enabled stable training within the constraints of our pilot study. LinkNet-based architectures have previously demonstrated competitive segmentation performance relative to conventional U-Net architectures and models incorporating multi-scale feature extraction, although architectures employing bottleneck layers, batch normalisation, and more advanced encoder designs have reported superior quantitative performance [26]. Similarly, a systematic evaluation of models across CT datasets of mixed-sex populations demonstrated superior performance of SegResNet and Attention U-Net across bladder, rectum, and femur compared to ResNet-based architectures [27]. The ResNet-34 backbone employed in the current model is limited by the absence of group normalisation, global contextual modelling, and 3D convolution operations. These limitations may reduce volumetric continuity and impair segmentation of structures in close anatomical proximity with poor soft tissue contrast, which are particularly relevant challenges in abdominal and pelvic radiotherapy. Early studies of cervical cancer auto-segmentation established the feasibility of DL-based target volume delineation, with fusion-network approaches reporting DSC values of approximately 0.82 for primary CTV volumes [28]. Subsequent studies employing 2D and 3D U-Net architectures have shown similar quantitative performance, with some studies demonstrating advantages for 3D models in post-operative cases [29, 30]. Single-centre studies have reported favourble performance using 3D U-Net, fusion network and VB-Net based architectures [31–37], with limited generalisability to external populations. More recently, Wu et al. developed a multi-task fusion network incorporating distance-guided attention mechanisms and reported DSC values of 84.67% and 77.51% on internal and external validation datasets, respectively, outperforming several contemporary U-Net based and commercial models [38].

We observed a DSC of 0.58 ± 0.12 (NSD 0.66 ± 0.1) for primary CTV in our study. Unlike other studies, pelvic vessels were explicitly contoured in the current study to enable margin generation for pelvic nodal volumes. We incorporated the common, external, internal and obturator regions into one pelvic nodal CTV and contoured the inguinal region as a separate CTV. We achieved a DSC 0.07 ± 0.15 (NSD 0.18 ± 0.2) for the pelvic vessels after training on female pelvic datasets and DSC 0.6 ± 0.1 (NSD 0.71 ± 0.14) and 0.77 ± 0.04 (NSD 0.76 ± 0.07) for pelvic nodal and inguinal CTV, respectively. Studies reporting both primary and pelvic nodal CTV volumes are limited. In the largest reported training-validation cohort (*n* = 2,254), Rhee et al. achieved DSC values of 0.86 ± 0.08 and 0.81 ± 0.03 for primary and pelvic nodal CTV, respectively [39]. For composite pelvic nodal volumes excluding the inguinal region, other studies have reported DSC values ranging from 0.77 to 0.88 and HD values of approximately 20–22 mm [32, 40, 41]. Auto-segmentation of pelvic nodal subvolumes has also been explored [40, 42, 43]. However, reports specifically evaluating inguinal nodal CTV remain limited, with reported DSC values ranging from 0.87 to 0.94 and HD values from 3.69 mm to 4.8 mm [42, 44]. Evaluation across overlap-, distance-, and surface-based metrics demonstrated reasonable volumetric and surface agreement (NSD 0.66–0.76), while the larger HD95 values suggest the presence of occasional outlier contours.

With respect to pelvic OARs, the highest DSC of 0.92 ± 0.03 (NSD 0.94 ± 0.04), 0.89 ± 0.03 (NSD 0.77 ± 0.12), and 0.88 ± 0.14 (NSD 0.87 ± 0.12) were obtained for femur, bowel bag and bladder, respectively. Compared with contemporary 3D U-Net and the CNN-based context aggregation approaches, DC demonstrated favourable performance for femur and bowel bag, exceeding the DSC reported by Wang et al. (0.88 and 0.86 ± 0.04) and Liu et al. (0.9 and 0.833 ± 0.035) [31, 45]. Similarly, bladder (16.03 mm ± 35.14) and rectum (22.41 mm ± 27.32) achieved lower HD95 values than those reported by a 3D transfer-learning model trained on prostate MRI datasets [46].

In the abdomen, DC achieved DSC of 0.83 ± 0.07 (NSD 0.84 ± 0.11) for stomach and 0.83 ± 0.11 (NSD 0.86 ± 0.11) for kidneys. Stomach performance exceeded that reported by Chung et al. (DSC 0.67 ± 0.27), while bowel bag performance (0.89 ± 0.03 [NSD 0.77(0.12]) was comparable (Chung et al., 0.9 ± 0.02), and superior to Tian et al. (DSC 87.53%). Kidney performance, however remained lower than that reported by Chung et al. (DSC 0.9) and Tian et al. (DSC 96%) highlighting the potential advantage of attention-based fusion architectures [40, 47].

Besides conventional overlap- and distance-based metrics, we incorporated NSD, which remains infrequently reported in pelvic auto-segmentation studies. Unlike DSC, which quantifies volumetric overlap, NSD evaluates agreement at the contour surface and therefore may better reflect the magnitude of edits required during clinical contour review [48, 49]. Across most structures in our study, the NSD values were comparable to or exceeded the corresponding DSC values, suggesting that geometric differences were often confined to limited boundary regions rather than representing contour failure. This may better reflect clinical contour review, where edits typically involve boundary refinement rather than complete re-delineation of structures. A summary of the DSC scores from key contemporary studies in comparison with those of the current study can be found in Supplementary File 2.

Clinical evaluation has become an essential component of auto-segmentation studies, as it reflects real-world clinical usability beyond the geometric measures. However, there is limited standardisation seen in clinical evaluation methodologies across the literature, including variations in observer numbers, scoring systems, and validation dataset numbers [34, 39, 40, 42, 50, 51]. Although we observed a high overall scores, particularly among internal experts, this was limited to only two validation cases and should be considered exploratory. Structures such as the kidneys, bladder, the femur and bowel bag demonstrated consistently high acceptance, mirroring their favourable geometric performance. In contrast, the duodenum, sigmoid, and anal canal were frequently identified as requiring substantial edits.

DC completed auto-segmentation of all target volumes and OARs in 5 s per dataset in a controlled laboratory setting, compared with a mean manual segmentation time of 77 min. However, these findings represent model inference time only, and prospective evaluation of clinician review, contour editing, DICOM transfer, and deployment infrastructure is necessary to determine the true impact on clinical workflow efficiency. This remains a planned evaluation in the future. Nevertheless, the magnitude of difference suggests considerable potential for workflow improvement following clinical implementation. Few studies have reported model inference times, which range from < 1 s to 11 s per dataset for 2D models and 9–11 s to 56–90 s for 3D models [30, 52]. A summary of all the cervical cancer studies that evaluated qualitative scoring and time-savings can be found in Supplementary File 2.

There is a dearth of contemporary auto-segmentation research originating from low- and middle-income countries (LMICs), with published work largely limited to breast and head-and-neck cancers [53–56]. In pelvic malignancies, experience from LMIC settings remains particularly sparse, with one study reporting on evaluation of auto-segmentation for prostate cancer OARs [57]. Given the substantial gap between the optimal radiotherapy utilisation rate (58.37%) and actual radiotherapy utilisation rate (28.5%) in India, with only 5.13% of cervical cancer cases receiving radiotherapy treatment [58], technologies that reduce contouring workload and improve planning efficiency may have considerable clinical value. Our study represents one of the first prospective evaluations of an locally developed DL-based auto-segmentation model for cervical cancer target volumes and abdominopelvic OARs. The favourable geometric performance and substantial reduction in contouring time support the feasibility of developing auto-segmentation solutions for cervical cancer radiotherapy in resource-constrained settings. Furthermore, the inclusion of pelvic nodal volumes, inguinal nodal regions, and a comprehensive range of abdominal and pelvic OARs may facilitate future adaptation of the model to extended-field radiotherapy, other gynaecological malignancies, and selected gastrointestinal and pelvic cancers.

The present study has several limitations. Firstly, DC was trained using limited datasets from a single institution and without external validation. Second, the slice thickness varied from 2 mm to 5 mm across the cohort. Variable slice thickness results in anisotropic voxel spacing and represents a methodological limitation of this pilot, as it introduces heterogeneity in z-axis resolution that may affect model learning. Although using local datasets as inputs potentially allows adaptation to commonly seen anatomical characteristics and institutional contouring practices, more rigorous preprocessing, including resampling to a fixed voxel size, may improve reliability and reproducibility. Thirdly, despite the application for cervical cancer radiotherapy, 31.6% of the training cohort for OAR comprised male pelvic CT datasets. The inclusion of male datasets was limited to augmentation of the OAR cohort, while targets were primarily from cervical cancer. Previous studies have also reported comparable segmentation performance between mixed-sex and sex-specific training approaches for pelvic nodal and OAR auto-segmentation [32, 43, 59]. Nevertheless, inclusion of mixed-sex data may introduce feature-learning noise, potentially affecting model generalisability to a female-only population. Fourth, the clinical acceptability evaluation was performed for only two validation datasets and the time required for clinician review and correction of auto-segmented contours was not prospectively recorded. Therefore, although the model inference time was substantially shorter than that of manual contouring, the true impact on end-to-end clinical workflow efficiency could not be quantified in the present study. Fifth, the 2D U-Net architectures have shown comparable or improved performance for the larger organs, such as the liver and kidneys in CT datasets, whereas more variable pelvic organs like bladder and prostate performed well in MRI datasets [60, 61]. Nevertheless, the failure analysis in our study revealed a lower performance for anatomically small, tortuous or poorly defined structures such as the anal canal and CBD. This may reflect the inherent limitations due to the lack of volume continuity and global contextual information in 2D LinkNet architecture. A formal explainability or interpretability analysis was not performed, as the model output consisted of segmentation masks rather than feature-based predictions.

## Conclusion

This study demonstrates the feasibility of a DL-based auto-segmentation model for cervical cancer radiotherapy using real-world clinical datasets. Favourable geometric performance was demonstrated across OARs and inguinal CTV with substantial time savings highlighting its potential utility in routine practice. Further validation in larger and external datasets, alongside dosimetric impact, clinical acceptability, clinical workflow integration, and measurement of efficiency gains, is warranted to establish its role in routine radiotherapy planning and clinical impact in resource-constrained settings.

## Supplementary Information

Supplementary Material 1.

Supplementary Material 2.

## Data Availability

The datasets generated during and/or analyzed during the current study are not publicly available due to institutional data privacy regulations but are available from the corresponding author on reasonable request.

## Abbreviations

OAR: Organ at Risk
DL: Deep Learning
CT: Computed Tomography
DSC: Dice Similarity Coefficient
JI: Jaccard Index
HD95: 95th Percentile Hausdorff Distance
ASSD: Average Symmetric Surface Distance
NSD: Normalized Surface Dice
TTI: Time to Treatment Initiation
OTT: Overall Treatment Time
RCR: Royal College of Radiologists
CNN: Convolutional Neural Network
NICE: National Institute for Health and Care Excellence
TPS: Treatment Planning System
DC: DEEP CONTOUR
CTVp: Clinical Target Volume Primary
CTVn_pelvis: Clinical Target Volume Pelvic Nodal Region
CTVn_inguinal: Clinical Target Volume Inguinal Nodal Region
CBD: Common Bile Duct
LMIC: Low–and Middle–Income Country
