# Supplementary material for: A prospective development and evaluation of a 2D convolutional neural network-based auto-segmentation model for cervical cancer radiotherapy
Source: J Egypt Natl Canc Inst. 2026 Jul 15;38:46. doi: 10.1186/s43046-026-00382-7 (PMC13373109; doi:10.1186/s43046-026-00382-7)
Supplement: Supplementary file 1 — Supplementary Material 1. [file 43046_2026_382_MOESM1_ESM.docx]

**Checklist for Artificial Intelligence in Medical Imaging (CLAIM): 2024 Update**

| Section / Topic | No. | Item | Page / Line | No | NA |
| --- | --- | --- | --- | --- | --- |
| TITLE / ABSTRACT |  |  |  |  |  |
|  | **1** | Identification as a study of AI methodology, specifying the category of technology used (e.g., deep learning) | **1/ 2-3** |  |  |
| ABSTRACT |  |  |  |  |  |
|  | **2** | Summary of study design, methods, results, and conclusions | **1-2/4-33** |  |  |
| INTRODUCTION |  |  |  |  |  |
|  | **3** | Scientific and/or clinical background, including the intended use and role of the AI approach | **2-4/40-81** |  |  |
|  | **4** | Study aims, objectives, and hypotheses | **4/92-95** |  |  |
| METHODS |  |  |  |  |  |
| *Study Design* | **5** | Prospective or retrospective study | **4/85** |  |  |
|  | **6** | Study goal | **4/92-93** |  |  |
| *Data* | **7** | Data sources | **4-5/85-86, 102-104** |  |  |
|  | **8** | Inclusion and exclusion criteria | **5/104-111** |  |  |
|  | **9** | Data pre-processing | **6/122-133** |  |  |
|  | **10** | Selection of data subsets | **8/180-185** |  |  |
|  | **11** | De-identification methods | **4-5/100-102, 104-107, 119-120** |  |  |
|  | **12** | How missing data were handled | **5/109-111** |  |  |
|  | **13** | Image acquisition protocol | **5/107-109** |  |  |
| *Reference Standard* | **14** | Definition of method(s) used to obtain reference standard | **5-6/111-115** |  |  |
|  | **15** | Rationale for choosing the reference standard | **5-6/113-115** |  |  |
|  | **16** | Source of reference standard annotations | **5/111-113** |  |  |
|  | **17** | Annotation of test set | **6/118-119** |  |  |
|  | **18** | Measures of inter- and intra-rater variability of features described by the annotators |  | **No** |  |
| *Data Partitions* | **19** | How data were assigned to partitions | **8/180-185** |  |  |
|  | **20** | Level at which partitions are disjoint | **8/182-184** |  |  |
| *Testing Data* | **21** | Intended sample size | **8/180-181** |  |  |

| Section / Topic | No. | Item | Page / Line | No | NA |
| --- | --- | --- | --- | --- | --- |
| *Model* | **22** | Detailed description of model | **6-7/135-148** |  |  |
|  | **23** | Software libraries, frameworks, and packages | **6-7/144-161** |  |  |
|  | **24** | Initialization of model parameters | **7/151-155** |  |  |
| *Training* | **25** | Details of training approach | **7/144-148, 155-164** |  |  |
|  | **26** | Method of selecting the final model | **7/163-64** |  |  |
|  | **27** | Ensembling techniques | **7/148** |  |  |
| *Evaluation* | **28** | Metrics of model performance | **7/161-170** |  |  |
|  | **29** | Statistical measures of significance and uncertainty | **8/166-176** |  |  |
|  | **30** | Robustness or sensitivity analysis |  | **No** |  |
|  | **31** | Methods for explainability or interpretability | **24/440-442** | **No** |  |
|  | **32** | Evaluation on internal data | **8/167-176** |  |  |
|  | **33** | Testing on external data |  | **No** |  |
|  | **34** | Clinical trial registration | **4/88** |  |  |
| RESULTS |  |  |  |  |  |
| *Data* | **35** | Numbers of patients or examinations included and excluded | **8/180-184** |  |  |
|  | **36** | Demographic and clinical characteristics of cases in each partition | **9/196-201. Table 1. Table 2** |  |  |
| *Model performance* | **37** | Performance metrics and measures of statistical uncertainty | **12-13/221-238. Table 3. Figure 4. 16-17/252 – 288. Table 4** |  |  |
|  | **38** | Estimates of diagnostic performance and their precision |  |  | **NA** |
|  | **39** | Failure analysis of incorrect results | **21/372-376, 24/437-440** |  |  |
| DISCUSSION |  |  |  |  |  |
|  | **40** | Study limitations | **23-24/416-442** |  |  |
|  | **41** | Implications for practice, including intended use and/or clinical role | **24/447-450** |  |  |
| OTHER INFORMATION |  |  |  |  |  |
|  | **42** | Provide a reference to the full study protocol or to additional technical details | **Title page** |  |  |
|  | **43** | Statement about the availability of software, trained model, and/or data | **Title page** |  |  |
|  | **44** | Sources of funding and other support; role of funders | **Title page** |  |  |

* Indicate page and/or line number for each checklist item that is present. NA = not applicable.
