# Supplementary material for: A prospective development and evaluation of a 2D convolutional neural network-based auto-segmentation model for cervical cancer radiotherapy
Source: J Egypt Natl Canc Inst. 2026 Jul 15;38:46. doi: 10.1186/s43046-026-00382-7 (PMC13373109; doi:10.1186/s43046-026-00382-7)
Supplement: Supplementary file 2 — Supplementary Material 2. [file 43046_2026_382_MOESM2_ESM.docx]

Table 1. Expert qualitative evaluation of autosegmentation for organs at risk in cervical cancer radiotherapy using a 4-point Likert scale.

| **Case I (internal experts)** | | | | |
| --- | --- | --- | --- | --- |
| **Structure** | **I** | **II** | **III** | **IV** |
| Stomach |  |  | 4(80.0%) | 1(20.0%) |
| Duodenum |  | 1(20.0%) | 4(80.0%) |  |
| Pancreas | 1(20.0%) | 4(80.0%) |  |  |
| Liver | 3(60.0%) | 2(40.0%) |  |  |
| Kidneys |  |  | 2(40.0%) | 3(60.0%) |
| Common bile duct |  |  |  | 5(100.0%) |
| Bowel bag (Abdominal) |  |  | 1(20.0%) | 4(80.0%) |
| Bowel bag (Pelvic) |  |  |  | 5(100.0%) |
| Bladder |  |  |  | 5(100.0%) |
| Sigmoid |  | 2(40.0%) | 3(60.0%) |  |
| Rectum |  |  |  | 5(100.0%) |
| Anal canal |  | 1(20.0%) | 3(60.0%) | 1(20.0%) |
| Femur |  |  |  | 5(100.0%) |
| Genitalia |  |  | 4(80.0%) | 1(20.0%) |
| **Case II (internal experts)** | | | | |
| **Structure** | **I** | **II** | **III** | **IV** |
| Stomach |  |  | 2(40.0%) | 3(60.0%) |
| Duodenum |  |  | 4(80.0%) | 1(20.0%) |
| Pancreas |  | 2(40.0%) | 3(60.0%) |  |
| Liver |  |  | 3(60.0%) | 2(40.0%) |
| Kidneys |  |  | 3(60.0%) | 2(40.0%) |
| Common bile duct |  |  |  | 5(100.0%) |
| Bowel bag (Abdominal) |  |  | 5(100.0%) |  |
| Bowel bag (Pelvic) |  |  | 2(40.0%) | 3(60.0%) |
| Bladder |  |  | 1(20.0%) | 4(80.0%) |
| Sigmoid |  |  | 2 (40.0%) | 3(60.0%) |
| Rectum |  | 1(20.0%) | 2 (40.0%) | 2 (40.0%) |
| Anal canal |  |  | 3(60.0%) | 2(40.0%) |
| Femur |  |  | 2(40.0%) |  |
| Genitalia |  |  | 2(40.0%) | 3(60.0%) |
|  | | | | |
| **Case I (external experts)** | | | | |
|  | **I** | **II** | **III** | **IV** |
| Stomach |  | 1(50.0%) | 1(50.0%) |  |
| Duodenum |  | 1(50.0%) | 1(50.0%) |  |
| Pancreas |  | 1(50.0%) | 1(50.0%) |  |
| Liver | 1(50.0%) | 1(50.0%) |  |  |
| Kidneys |  |  | 2(100.0%) |  |
| Common Bile duct |  | 2(100.0%) |  |  |
| Bowel bag (Abdominal) |  | 1(50.0%) | 1(50.0%) |  |
| Bowel bag (Pelvic) |  |  | 2(100.0%) |  |
| Bladder |  |  | 2(100.0%) |  |
| Sigmoid |  |  | 2(100.0%) |  |
| Rectum |  |  | 2(100.0%) |  |
| Anal canal |  | 1(50.0%) | 1(50.0%) |  |
| Femur |  |  |  | 2(100.0%) |
| Genitalia |  | 1(50.0%) | 1(50.0%) |  |
| **Case II (external experts))** | | | | |
| **Structure** | **I** | **II** | **III** | **IV** |
| Stomach |  |  | 2(100.0%) |  |
| Duodenum |  |  | 2(100.0%) |  |
| Pancreas |  | 1(50.0%) | 1(50.0%) |  |
| Liver |  |  | 2(100.0%) |  |
| Kidneys |  |  | 1(50.0%) | 1(50.0%) |
| Common bile duct | 1(50.0%) | 1(50.0%) |  |  |
| Bowel bag (Abdominal) |  |  | 2(100.0%) |  |
| Bowel bag (Pelvic) |  |  | 1(50.0%) | 1(50.0%) |
| Bladder |  |  | 1(50.0%) | 1(50.0%) |
| Sigmoid |  |  | 1(50.0%) | 1(50.0%) |
| Rectum |  |  | 1(50.0%) | 1(50.0%) |
| Anal canal |  |  | 1(50.0%) | 1(50.0%) |
| Femur |  |  | 1(50.0%) | 1(50.0%) |
| Genitalia |  | 1(50.0%) |  | 1(50.0%) |

Likert scores for two independent test cases (Case I and Case II). Experts rated each structure using a 4-point Likert scale based on clinical acceptability: I = unacceptable, major revision required; II = unacceptable, minor revision required; III = acceptable with minor edits; IV = clinically acceptable without revision. Values represent the number of experts assigning each score, with percentages shown in parentheses. No observers were involved in model development.

Table 2. Comparison of Dice Similarity Coefficient (DSC) values for deep learning-based autosegmentation of target volumes and organs at risk in cervical cancer radiotherapy across contemporary studies and the current study.

| Structure | Wen et al[1] | Chung. et al[2] | Ding et. al[3] | Xiao et al[4] | Wang et al[5] | Chen et al[6] | Peng et al[7] | Current study |
| --- | --- | --- | --- | --- | --- | --- | --- | --- |
| GTV primary | - | - | - | - | - | - | 0.71 | NA |
| CTV primary | - | 0.76 | 0.83 | 0.91 | 0.85 | - | 0.82 | 0.58 ± 0.12 |
| CTV pelvic lymph nodal region | - | 0.77 | - | - | - | - | 0.81 | 0.6 ± 0.1 |
| CTV inguinal lymph nodal region | 0.94 | - | - | - | - | - | - | 0.77 ± 0.04 |
| Bowel bag | - | 0.9 | 0.79 (small bowel) | 0.86(small bowel) | 0.74(small bowel, 0.81 (colon) | 0.82 (colon) | - | 0.89 ± 0.03 |
| Bladder | - | 0.88 | 0.94 | 0.91 | 0.93 | 0.95 | - | 0.88 ± 0.14 |
| Sigmoid | - | - | - | - | - | - | - | 0.54 ± 0.17 |
| Rectum | - | 0.86 | - | - | - | - | - | 0.75 ± 0.16 |
| Femur | - | 0.89 | - | - | - | - | - | 0.92 ± 0.03 |
| Genitalia | - | - | - | - | - | - | - | 0.81 ± 0.09 |
| Anal canal | - | - | - | - | - | - | - | 0.27 ± 0.23 |
| Pelvic vessels | - | - | - | - | - | - | - | 0.07 ± 0.15 |
| Stomach | - | - | - | - | - | - | - | 0.83 ± 0.07 |
| Duodenum | - | - | - | - | - | - | - | 0.55 ± 0.16 |
| Pancreas | - | - | - | - | - | - | - | .15 ± .26 |
| CBD | - | - | - | - | - | - | - | .01 ± 0.11 |
| Liver | - | - | - | - | - | - | - | .73 ± .21 |
| Kidneys | - | - | - | - | - | - | - | .83 ± .11 |

DSC values from published studies are reported as provided in the respective manuscripts. Values from the current study are presented as mean ± standard deviation. DSC ranges from 0 to 1, with higher values indicating greater spatial overlap between manual and automated contours. “–” indicates that the structure was not evaluated or not reported in the corresponding study.

Table 3. Summary of cervical cancer studies, the numer of datasets, reference standard used and methods of clinical evaluation and time-saving incorporated

| **Study** | **Model** | **Datasets** | **Ground truth** | **Clinical Evaluation** | **Time-saving** |
| --- | --- | --- | --- | --- | --- |
| Wang et al.,(2020)[8,9] | Study 1; Novel 3D-CNN  Novel 2D-CNN ‘Conv2d’ model | Study 1; Single center.  125 datasets - 75 training, 25 validation and test.  Study 2; 300 datasets for trainging and validation from multiple centers. No information on distribution.  75 test datasets | Study 1; One senior physician over 20 years of exp.  Study 2; One junior oncologist’s contours reviewed by one expert | Study 1; Not done  Study 2; Not done | Study 1; Average time of autosegmentation per case - 2 min average. Manually time taken - 90 min average  Study 2; Not done |
| Sartor et al., (2020)[10] | Novel 3D-CNN | 65 datasets (80% training, 20% validation). 10 Tests datasets | Original clinical contours by senior residents, reviewed by a senior oncologist. | One oncologist over 20 years of exp on a 4-point Likert scale. Each structure of each dataset. | Not done |
| Rigaud et al., (2021)[11] | One publicly available and commerical model - 2D DeepLabV3+ and 3D U-Net | Multicentric. 166 Training, 26 validation, 62 and 30, internal and external test sets, respectively. | One observer per center (>5 years and >8 years of exp, repectively).  Original clinical contours in internal test set. Observer from other center contoured on external test datasets | Not done | Manual time of 1-2 hours.  Autosegmentation time not assessed. |
| Liu et al., (2020, 2021)[12–14] | Study 1; Novel U-Net. Compared with conventional U-Net architecture | Study 1; Single center.  101 datasets – 77 training, 14 datasets each in validation and test sets | Study 1; Original clinical contours verified by committee of eight experts | Study 1; Not done | Study 1; Processing time of one slice by model – 0.06 sec  No comparison with manual segmentation time made. |
|  | Study 2; Novel DpnUNet (2.5D). Compared with Multiple models | Study 2; Single center. 210, five-fold cross validation. Test set of 27 patients | Study 2; Original clinical contours. | Study 2; Three oncologists did blinded evaluation of two model ouputs, using 4-point Likert scale. | Study 2; Average time of autosegmentation per case - 15 sec and time for manual revision assessed – 10.37 +- 3.42 min per test case.  Total workflow time of 15 min. Manual time not |
|  | Study 3; DpnUnet with ResNet-10 discriminator | Study 3; Trained and tested on 237 patients in a previous study.  New validation of CTV alone in 20 patient dataset | Study 3; Validation set contours reviewed by two senior oncologists followed by review by committee of eight oncologists | Study 3; 20 random slices of 10 random cases evaluated by nine experts from six centers, on a 3-point likert scale and Turing’s imitation test  Evaluated at 2-week intervals. | Study 3; Not done |
| Rhee et al., (2020)[15] | Novel Inception-ResNet-V2. Novel 2D FCN-8s. Novel 3D V-Net model | Base model – trained on 406 datasets.  Study datasets - 2254 datasets in total, divided into 80:20 for training and validation (210 additional datasets from a segmentation challenge set).  370 – validation set for bony structures. 140 internal and 30 external test sets(multicentric). | Four observers – curated or manually contoured datasets. Bony structures manually contoured by medical physicists, and reviewed by oncologists | Unknown number of observers scored on 3-point likert scale | Not done |
| Shi et al., (2021)[16] | 3D U-Net with additional strategies. Compared multiple models | 315 radical cases, 147 post-operative cases. | Two junior radiologist observers. | Not done | Not done |
| Ju et al., (2021)[17] | Novel 3D Dense-V-Network. Compared with multiple models. | 190 total datasets (123 post-operative). Randomly divided into 160 training and 30 test. | One attending physician. | Not done | Not done |
| Ma et al., (2021)[18,19] | Study 1; VB-Net  Study 2; 3D VB-Net. Compared with DL-based method with other non DL-based registration methods | Study 1; 157, 20, 23 for training, validation and test of radical cases, respectively.  272, 30, 33 for training, validation and test of post-operative cases, respectively.  Study 2; Definitive – Training and validation set – 23; test set – 7. Post-operative - Training and validation set – 69; test set – 8. | Study 1; Senior oncolgists approved contours.  No information on number of observers or reviewers  Study 2; Multiple, unknown number of observers. | Study 1; Nine oncologists of varying experience evaluated on 4-point likert scale.  Study 2; Not done | Study 1; Autosegmentation under one sec.  Time for manual contouring of each oncologist and AI-assisted contours ranged from 9-48 minutes depending on type of CTV being contoured.  No formal comparison made  Study 2; Not done |
| Chang et al., (2021)[20] | Novel 3D U-Net. Pre-trained and raw versions of model compared and assessed in adaptive training | Multicentric.  Five groups of 80 datasets from three centers. | Two observers for two dataset groups. Multiple observers for rest of the groups. | Not done | Only model training time assessed. |
| Nie et al., (2022)[21] | DenseNet (U-Net) and Novel 3D SegNet in a two stage framework. | 121, 22, and 63 post-operative datasets for training, validation and test, respectively. 20 additional prospective cases | Originial clinical contours curated by three observers, reviewed by experts. | Three observers evaluated on 6-point likert scale. | Average time for AI-assisted contouring - 9.54 ± 2.42 min and manual contouring -30.95 ± 15.24 min |
| Chen et al., (2022)[6] | 3D U-Net | 127 training (65 supine and 62 prone position). 22 test (13 supine and 9 prone) | Three observers. Contours further reviewed by experts. | Not done | Not done |
| Xiao et al., (2022)[4] | Novel 3D ‘RefineNetPlus3D’ model. Compared with other models | Post-operative cases randomly divided into 251 training and 31 validation and test datasets | Two senior oncologists of > 10 years exp as observers | Not done | Autosegmentation and computing time reported.  Autosegmentation time of 2D models were <1 sec, while for 3D models ranged from 9-11 sec |
| Tian et al., (2023)[22] | Novel parallel-path attention fusion network (PPAF-net) with custom modules. Compared with multiple models. | 276 total datasets. No information regarding validation and test datasets. | One observer delineated contours, reviewed by three-five experts | Not done | Not done |
| Chung et al., (2023)[2] | U-Net model. Not known if 2D or 3D | 165 for training and 15 for validation set | Single expert oncologist | Turing’s imitation test on random slices from validation set performed in the form of questionnaire by unknown number of observers  Evaluated on a 10-point likert scale by unknown number of observers | Time taken by 10 oncologists’ manual delineation from six centers compared with autosegmentation  Time to edit autocontours by multiple observers assesed |
| Jiang et al., (2024) [23] | Novel 3D-CNN. Separate model for each subgroup of CTV nodal volume. | 152 post-operative datasets. 96, 36, 20 for training, validation and test, respectively. | One junior oncologist’s contours reviewed by expert oncologist of >15 years exp when required. | Not done | Not done |
| Wen et al., (2024)[24] | Novel 2D cascaded multi-heads U-net (CMU-net). Compared with other models. | 120 for training and validation, including rectal and prostate datasets.  40 test datasets  Cervical cancer only consisted of 22% and 25% of trainin-validation and test cohorts, respectively. | Two observers, verified by one expert oncologist | Multiple experts evaluated on a 7-point likert scale in terms of number of slices requiring edits. | Not done |
| Peng et al., (2024)[7] | 3D U-Net. Multi-decoder and semi-supervised learning (MDSSL) method used  Separate models for GTV and CTV used | 31 datasets randomly divided into 4:1 of training and validation sets  (Equally divided radical and post-opearative cases). 40 test datasets.  Additional 59 diagnostic radical(26) and post-operative(33) CT scans used for semi-supervised model  Additional validation on 9 CBCTs of cervical cancer for online ART workflow | Unknown number and expertise of observers | Not done | Time for editing autosegmentation reported in the CBCT images |
| Rayn et al., (2024)[25] | Commercial CNN - Deep image-to-image network (DI2IN). Not known if 2D or 3D | No information of distribution of training, validation and test sets.  Gender distributed datasets. 37 cervical cancer cases (5 post-operative). Various other pelvic cancers used. | Not reported | One expert evaluated on 4-point likert scale in terms of requiring edits  Graded on a 4-point likert scale depending on previous scale scores | Not done |
| Douglas et al., (2024)[26] | Novel Inception-ResNet-V2 | 39 cervical cancer datasets. No information on training-validation-test distribution | Reported dose to manual contour considered as ground truth (GT) | Not done | Not done |
| Bordigoni et al., (2024)[27] | Commerical Mvision and Limbus models. Compared with ‘Elekta ABAS’ and ‘Elekta Admire’ tools. | Single center. No explicit training carried out. 20 test datasets for cervical and prostate cancer cases each. | Two expert observers | Two cervical cancer experts and one prostate cancer expert evaluated five cases on a 5-point likert scale. | Autosegmentaton time per structure in five random cervical and prostate cancer cases each |
| Wu et al., (2025) [28] | Novel ResCANet. Not known if 2D or 3D. Compared with other models | 189 for training and 47 for 5-fold cross internal validation. External validation on 54 endometrial datasets | Original clinical contours refiewed by one observer of >20 year exp. | One expert evaluated on a scale of time needed to revise AI generated contours and assigned acceptance scores | Mean time of slice-by-slice manual delineation of 1 hour  Time required to revise autosegmentation assessed |
| Yucheng et., (2025)[29] | Segment anything model (SAM) and recursive learning (RL) based architecure. Res-Net50 backbone.  Used deep reinforement learning (DRL) and DL strategies. Compared with commerical model. | 122 and 28 datasets divided inot training and test sets, respectively. | One expert with <10 years exp, verfied by expert of >15 years exp | Not done | Autosegmentation time of 30 sec per case  Not compared with manual contouring time. |
| Sun et al., (2025)[30] | Three models used; One Machine learning (ML) model based on support vector machine (SVM) approach and two DL based CNN models | Randomized to obtain 588 fractions from 21 patients for training and test through 5-fold cross validation (4:1 dvision)  Independent test set of 83 fractions from 3 remaining patients | AI-generated contours, verified by one observer | Three observers evaluated criteria of ‘trigger’ and defined dose-deviation for the clinical evaluation of plans. | Time taken for autosegmented volume editing reported.  No formal comparison of time-saving. |
| Xu et al., (2026)[31] | Novel 3D nnU-Net model, made publicly available.  Three versions compared | 177, 41 and 300 for training, internal and external validation (5-fold cross), respectively (Definitive and pos-operative included)  84 endometrial cases for external validation. | Original clinical contours by junior oncologists, verified by senior oncologists.  All contours verified by a committee of three experts | Blinded review by a committee of three experts on a 4-point likert scale. | Inference time of models reported.  No formal comparison of time-saving. |

**References**

1. Wen F, Zhou J, Chen Z, Dou M, Yao Y, Wang X, et al. Efficient application of deep learning-based elective lymph node regions delineation for pelvic malignancies. Med Phys. 2024 Oct;51(10):7057–66. doi:10.1002/mp.17330 PubMed PMID: 39072765.

2. Chung SY, Chang JS, Kim YB. Comprehensive clinical evaluation of deep learning-based auto-segmentation for radiotherapy in patients with cervical cancer. Front Oncol. 2023 Apr 28;13. doi:10.3389/fonc.2023.1119008

3. Ding Y, Chen Z, Wang Z, Wang X, Hu D, Ma P, et al. Three-dimensional deep neural network for automatic delineation of cervical cancer in planning computed tomography images. J Appl Clin Med Phys. 2022;23(4):e13566. Located at: Medline. doi:10.1002/acm2.13566

4. Xiao C, Jin J, Yi J, Han C, Zhou Y, Ai Y, et al. RefineNet-based 2D and 3D automatic segmentations for clinical target volume and organs at risks for patients with cervical cancer in postoperative radiotherapy. J Appl Clin Med Phys. 2022;23(7):e13631. Located at: CN-02424369. doi:10.1002/acm2.13631

5. Wang J, Chen Y, Xie H, Luo L, Tang Q. Evaluation of auto-segmentation for EBRT planning structures using deep learning-based workflow on cervical cancer. Sci Rep. 2022 Aug 11;12(1):13650. doi:10.1038/s41598-022-18084-0 PubMed PMID: 35953516; PubMed Central PMCID: PMC9372087.

6. Chen A, Chen F, Li X, Zhang Y, Chen L, Chen L, et al. A Feasibility Study of Deep Learning-Based Auto-Segmentation Directly Used in VMAT Planning Design and Optimization for Cervical Cancer. Front Oncol. 2022;12((Chen A.; Chen L.; Chen L., chenlx@sysucc.org.cn; Zhu J., zhujh@sysucc.org.cn) Department of Radiation Oncology, State Key Laboratory of Oncology in South China, Collaborative Innovation Center for Cancer Medicine, Sun Yat-sen University Cancer Center, Guangzhou, China). Located at: Embase. doi:10.3389/fonc.2022.908903

7. Peng H, Liu T, Li P, Yang F, Luo X, Sun X, et al. Automatic delineation of cervical cancer target volumes in small samples based on multi-decoder and semi-supervised learning and clinical application. Sci Rep. 2024 Nov 6;14(1). Located at: WOS:001350513800011. doi:10.1038/s41598-024-78424-0

8. Wang Z, Chang Y, Peng Z, Lv Y, Shi W, Wang F, et al. Evaluation of deep learning-based auto-segmentation algorithms for delineating clinical target volume and organs at risk involving data for 125 cervical cancer patients. J Appl Clin Med Phys. 2020 Dec;21(12):272–9. doi:10.1002/acm2.13097 PubMed PMID: 33238060; PubMed Central PMCID: PMC7769393.

9. Wang J, Chen Y, Xie H, Luo L, Tang Q. Evaluation of auto-segmentation for EBRT planning structures using deep learning-based workflow on cervical cancer. Sci Rep. 2022;12(1):13650. Located at: Medline. doi:10.1038/s41598-022-18084-0

10. Sartor H, Minarik D, Enqvist O, Ulén J, Wittrup A, Bjurberg M, et al. Auto-segmentations by convolutional neural network in cervical and anorectal cancer with clinical structure sets as the ground truth. Clin Transl Radiat Oncol. 2020;25:37–45. Located at: Scopus. doi:10.1016/j.ctro.2020.09.004

11. Rigaud B, Anderson BM, Yu ZH, Gobeli M, Cazoulat G, Söderberg J, et al. Automatic Segmentation Using Deep Learning to Enable Online Dose Optimization During Adaptive Radiation Therapy of Cervical Cancer. Int J Radiat Oncol Biol Phys. 2021 Mar 15;109(4):1096–110. doi:10.1016/j.ijrobp.2020.10.038 PubMed PMID: 33181248.

12. Liu Z, Liu X, Xiao B, Wang S, Miao Z, Sun Y, et al. Segmentation of organs-at-risk in cervical cancer CT images with a convolutional neural network. Phys Med. 2020;69((Liu Z.; Liu X.; Miao Z.; Sun Y.; Zhang F.) Department of Radiation Oncology, Peking Union Medical College Hospital, Chinese Academy of Medical Sciences&Peking Union Medical College, Beijing, China):184–91. doi:10.1016/j.ejmp.2019.12.008

13. Liu Z, Liu X, Guan H, Zhen H, Sun Y, Chen Q, et al. Development and validation of a deep learning algorithm for auto-delineation of clinical target volume and organs at risk in cervical cancer radiotherapy. Radiother Oncol. 2020;153((Liu Z., liuzk2009@126.com; Liu X., lxpumch@163.com; Guan H., guanhui@pumch.cn; Zhen H., zhenhongnan@pumch.cn; Sun Y., sunyuliang94@sina.com; Qiu J., qiujie@pumch.cn) Department of Radiation Oncology, Peking Union Medical College Hospital, Chinese Academy of Medical Sciences&Peking Union Medical College, Beijing, China):172–9. Located at: Embase. doi:10.1016/j.radonc.2020.09.060

14. Liu Z, Chen W, Guan H, Zhen H, Shen J, Liu X, et al. An Adversarial Deep-Learning-Based Model for Cervical Cancer CTV Segmentation With Multicenter Blinded Randomized Controlled Validation. Front Oncol. 2021;11:702270. doi:10.3389/fonc.2021.702270 PubMed PMID: 34490103; PubMed Central PMCID: PMC8417437.

15. Rhee DJ, Jhingran A, Rigaud B, Netherton T, Cardenas CE, Zhang L, et al. Automatic contouring system for cervical cancer using convolutional neural networks. Med Phys. 2020 Nov;47(11):5648–58. doi:10.1002/mp.14467 PubMed PMID: 32964477; PubMed Central PMCID: PMC7756586.

16. Shi J, Ding X, Liu X, Li Y, Liang W, Wu J. Automatic clinical target volume delineation for cervical cancer in CT images using deep learning. Med Phys. 2021 Jul;48(7):3968–81. doi:10.1002/mp.14898 PubMed PMID: 33905545.

17. Ju Z, Guo W, Gu S, Zhou J, Yang W, Cong X, et al. CT based automatic clinical target volume delineation using a dense-fully connected convolution network for cervical Cancer radiation therapy. BMC Cancer. 2021 Mar 8;21(1):243. doi:10.1186/s12885-020-07595-6 PubMed PMID: 33685404; PubMed Central PMCID: PMC7938586.

18. Ma CY, Zhou JY, Xu XT, Qin SB, Han MF, Cao XH, et al. Clinical evaluation of deep learning-based clinical target volume three-channel auto-segmentation algorithm for adaptive radiotherapy in cervical cancer. BMC Med Imaging. 2022 Jul 9;22(1):123. doi:10.1186/s12880-022-00851-0 PubMed PMID: 35810273; PubMed Central PMCID: PMC9271246.

19. Ma CY, Zhou JY, Xu XT, Guo J, Han MF, Gao YZ, et al. Deep learning-based auto-segmentation of clinical target volumes for radiotherapy treatment of cervical cancer. J Appl Clin Med Phys. 2022;23(2):e13470. doi:10.1002/acm2.13470

20. Chang Y, Wang Z, Peng Z, Zhou J, Pi Y, Xu XG, et al. Clinical application and improvement of a CNN-based autosegmentation model for clinical target volumes in cervical cancer radiotherapy. J Appl Clin Med Phys. 2021 Nov;22(11):115–25. doi:10.1002/acm2.13440 PubMed PMID: 34643320; PubMed Central PMCID: PMC8598149.

21. Nie S, Wei Y, Zhao F, Dong Y, Chen Y, Li Q, et al. A dual deep neural network for auto-delineation in cervical cancer radiotherapy with clinical validation. Radiat Oncol Lond Engl. 2022 Nov 15;17(1):182. doi:10.1186/s13014-022-02157-5 PubMed PMID: 36380378; PubMed Central PMCID: PMC9667653.

22. Tian M, Wang H, Liu X, Ye Y, Ouyang G, Shen Y, et al. Delineation of clinical target volume and organs at risk in cervical cancer radiotherapy by deep learning networks. Med Phys. 2023 Oct;50(10):6354–65. doi:10.1002/mp.16468 PubMed PMID: 37246619.

23. Jiang X, Zhang S, Fu Y, Yu H, Tang H, Wu X. Assembling High-quality Lymph Node Clinical Target Volumes for Cervical Cancer Radiotherapy using a Deep Learning-based Approach. Curr Med Imaging. 2024;20((Jiang X.; Fu Y., ychfu@hotmail.com; Yu H.; Tang H.) Department of Radiotherapy Physics&Technology Center, Cancer Center, West China Hospital Sichuan University, Sichuan Province, Chengdu, China). Located at: Embase. doi:10.2174/1573405620666230915125606

24. Wen F, Zhou J, Chen Z, Dou M, Yao Y, Wang X, et al. Efficient application of deep learning-based elective lymph node regions delineation for pelvic malignancies. Med Phys. 2024;51(10):7057–66. doi:10.1002/mp.17330

25. Rayn K, Gokhroo G, Jeffers B, Gupta V, Chaudhari S, Clark R, et al. Multicenter Study of Pelvic Nodal Autosegmentation Algorithm of Siemens Healthineers: Comparison of Male Versus Female Pelvis. Adv Radiat Oncol. 2024;9(2). Located at: Scopus. doi:10.1016/j.adro.2023.101326

26. Douglas R, Olanrewaju A, Mumme R, Zhang L, Beadle BM, Court LE. Evaluating automatically generated normal tissue contours for safe use in head and neck and cervical cancer treatment planning. J Appl Clin Med Phys. 2024 Jul;25(7):e14338. doi:10.1002/acm2.14338 PubMed PMID: 38610118; PubMed Central PMCID: PMC11244666.

27. Bordigoni B, Trivellato S, Pellegrini R, Meregalli S, Bonetto E, Belmonte M, et al. Automated segmentation in pelvic radiotherapy: A comprehensive evaluation of ATLAS-, machine learning-, and deep learning-based models. Phys Medica PM Int J Devoted Appl Phys Med Biol Off J Ital Assoc Biomed Phys. 2024 Sep;125:104486. doi:10.1016/j.ejmp.2024.104486 PubMed PMID: 39098106.

28. Wu Z, Wang D, Xu C, Peng S, Deng L, Liu M, et al. Clinical target volume (CTV) automatic delineation using deep learning network for cervical cancer radiotherapy: A study with external validation. J Appl Clin Med Phys. 2025 Jan;26(1):e14553. doi:10.1002/acm2.14553 PubMed PMID: 39401180; PubMed Central PMCID: PMC11712972.

29. Yucheng L, Lingyun Q, Kainan S, Yongshi J, Wenming Z, Jieni D, et al. Development and validation of a deep reinforcement learning algorithm for auto-delineation of organs at risk in cervical cancer radiotherapy. Sci Rep. 2025 Feb 25;15(1):6800. doi:10.1038/s41598-025-91362-9 PubMed PMID: 40000766; PubMed Central PMCID: PMC11861648.

30. Sun S, Gong X, Cheng S, Cao R, He S, Liang Y, et al. Fully Automated Online Adaptive Radiation Therapy Decision-Making for Cervical Cancer Using Artificial Intelligence. Int J Radiat Oncol Biol Phys. 2025 Jul 15;122(4):1012–21. doi:10.1016/j.ijrobp.2025.04.012 PubMed PMID: 40252932.

31. Xu B, Liu J, Fang M, Zhu H, Zhang Y, Zhang H, et al. Multicenter deep learning-based automatic delineation of CTV and PTV in uterine malignancy CT imaging. Radiother Oncol J Eur Soc Ther Radiol Oncol. 2026 Jan;214:111212. doi:10.1016/j.radonc.2025.111212 PubMed PMID: 41120056.
